# Characterization of Third Generation Cephalosporin- and Carbapenem-Resistant *Aeromonas* Isolates from Municipal and Hospital Wastewater

**DOI:** 10.3390/antibiotics12030513

**Published:** 2023-03-03

**Authors:** Sara Drk, Ana Puljko, Mia Dželalija, Nikolina Udiković-Kolić

**Affiliations:** 1 Division for Marine and Environmental Research, Ruđer Bošković Institute, Bijenička 54, 10 002 Zagreb, Croatia; 2 Department of Biology, Faculty of Science, University of Split, Ruđera Boškovića 33, 21 000 Split, Croatia

**Keywords:** *Aeromonas*, hospital wastewater, municipal wastewater, ESBL, AmpC, carbapenemase, plasmid transfer

## Abstract

Antibiotic resistance (AR) remains one of the greatest threats to global health, and *Aeromonas* species have the potential to spread AR in the aquatic environment. The spread of resistance to antibiotics important to human health, such as third-generation cephalosporins (3GCs) and carbapenems, is of great concern. We isolated and identified 15 cefotaxime (3GC)- and 51 carbapenem-resistant *Aeromonas* spp. from untreated hospital and treated municipal wastewater in January 2020. The most common species were *Aeromonas caviae* (58%), *A. hydrophila* (17%), *A. media* (11%), and *A. veronii* (11%). Almost all isolates exhibited a multidrug-resistant phenotype and harboured a diverse plasmidome, with the plasmid replicons ColE, IncU, and IncR being the most frequently detected. The most prevalent carbapenemase gene was the plasmid-associated *bla*_KPC-2_ and, for the first time, the *bla*_VIM-2_, *bla*_OXA-48,_ and *bla*_IMP-13_ genes were identified in *Aeromonas* spp. Among the 3GC-resistant isolates, the *bla*_GES-5_ and *bla*_MOX_ genes were the most prevalent. Of the 10 isolates examined, three were capable of transferring carbapenem resistance to susceptible recipient *E. coli*. Our results suggest that conventionally treated municipal and untreated hospital wastewater is a reservoir for 3GC- and carbapenem-resistant, potentially harmful *Aeromonas* spp. that can be introduced into aquatic systems and pose a threat to both the environment and public health.

## 1. Introduction

Antibiotic resistance (AR) is undoubtedly one of the greatest threats to global health [1]. In addition to the issue of AR in the clinical setting, there has been increasing interest in recent years in the role that the environment, particularly wastewater, plays in the maintenance and spread of AR. Due to the multi-faceted nature of AR, a One Health approach that incorporates interactions between the human, animal, and environmental microbiome is needed to fully understand the spread of AR [1]. Continuous identification, assessment and monitoring of AR hotspots is incredibly important for controlling and preventing the spread of AR. Hospital wastewater assessment is critical because hospital sewage is a major contributor of antibiotic-resistant bacteria (ARB) and antibiotic-resistance genes (ARGs) to wastewater systems and, subsequently, to the environment [2,3]. Another well-known AR hotspot is wastewater treatment plants (WWTPs), where bacterial populations are reduced many times over, but which still discharge large amounts of ARB into water bodies on a daily basis [4]. One of the predominant genera in treated wastewater is *Aeromonas* [5,6], which are used as bacterial indicators of environmental AR [6,7,8].

Members of the genus *Aeromonas* are gram-negative bacilli that are ubiquitous in aquatic environments, including freshwater, seawater, and sewage [9,10,11]. Some *Aeromonas* species, particularly *Aeromonas salmonicida*, *Aeromonas hydrophila*, and *Aeromonas veronii* are known fish pathogens [11]. However, *Aeromonas* species are also considered as potential human pathogens causing various diseases such as gastroenteritis, respiratory, blood and skin infections [12]. These diseases are mainly caused by four species, *Aeromonas caviae*, *Aeromonas dhakensis*, *A. hydrophila*, and *A. veronii* [13].

The rapid increase of AR in *Aeromonas* spp. is of serious concern [5,6]. Of particular concern is the increasing resistance of *Aeromonas* species to antibiotics of critical importance to human health, such as third-generation cephalosporins (3GCs) and carbapenems [3,14,15]. The 3GCs are generally used to treat infections caused by gram-negative bacteria. However, the increasing prevalence of resistance to these antibiotics mediated by extended-spectrum β-lactamases (ESBLs) has led to the increasing use of carbapenems as last-resort antibiotics for the treatment of these infections. The most common mechanism of resistance to carbapenems in gram-negative bacteria is the production of carbapenemases, which confer resistance to virtually all β-lactams. Carbapenem resistance in *Aeromonas* spp. is usually mediated by the “carbapenem-hydrolyzing *Aeromonas*” metallo-β-lactamase (CphA), which is encoded by the *cphA* gene on the chromosome [16,17]. Other clinically relevant and plasmid-associated carbapenemase genes such as *bla*_KPC-2_, *bla*_VIM-1_, *bla*_IMP-4_, *bla*_NDM-1_ and *bla*_OXA-830_ have also been detected in *Aeromonas* spp. both in the chromosome and on plasmids [3,18,19,20,21,22,23,24,25,26]. In addition, several plasmid-associated ESBL genes have also been found in ESBL-producing *Aeromonas* spp. such as *bla*_CTX-M-15_, *bla*_SHV-12_, *bla*_PER-1_, *bla*_FOX-2_, *bla*_VEB-9_ and *bla*_GES-5_, [10,14,27,28,29]. Therefore, *Aeromonas* spp. in the environment may serve as a reservoir for genes conferring resistance to antibiotics, which are vital in human medicine. Characterization of environmental *Aeromonas* strains is, therefore, critical for understanding AR from a One Health perspective.

To date, few studies have investigated the characteristics of 3GC- and carbapenem-resistant *Aeromonas* spp. from wastewater matrices [3,14,30]. Therefore, the aim of this study was to isolate and characterize carbapenemase- and ESBL-producing *Aeromonas* spp. from untreated hospital and treated municipal wastewater from the Zagreb WWTP. The AR profile against a number of clinically important antibiotics and the presence of priority ARGs (ESBL, carbapenemase) and plasmid replicon types in these isolates were analyzed by phenotypic and genotypic tests. In addition, we investigated the ability of selected carbapenemase-producing isolates to transfer their carbapenem-resistant phenotype to a β-lactam-susceptible *Escherichia coli*, and transferable plasmids were analyzed for carbapenemase genes and plasmid replicon types.

## 2. Results

### 2.1. Identification, AR Profiles and ß-Lactamase Production in Aeromonas isolates

**Figure 1:**
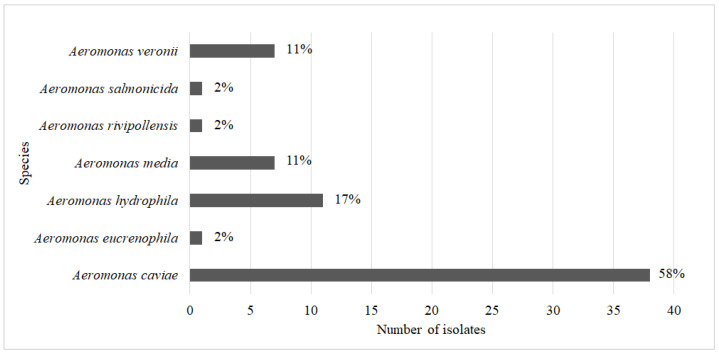
Number of isolates of each specific *Aeromonas* species.

A total of 66 isolates of the genus *Aeromonas* were isolated from municipal wastewater (*n* = 59) and hospital wastewater (*n* = 7). Of these, fifteen isolates were selected on selective media with cefotaxime (representative of 3GCs) and fifty one isolates were selected on media with carbapenems. Fifty isolates were identified to the species level using matrix-assisted laser desorption ionization–time-of-flight mass spectrometry (MALDI-TOF MS), and the other sixteen isolates, which could only be identified to the genus level using MALDI-TOF MS, were identified to the species level using their 16S rRNA gene sequences. The most prevalent species were *A. caviae* with 38 isolates (58%), followed by *A. hydrophila* with 11 isolates (17%), *Aeromonas media* and *A. veronii* with 7 isolates each (11%), and *Aeromonas eucrenophila*, *Aeromonas rivipollensis*, and *A. salmonicida* with only 1 isolate each (Figure 1).

To compare the accuracy of the MALDI-TOF MS method in identifying *Aeromonas* at the species level, 16S rRNA sequencing of 35 *Aeromonas* spp. carrying carbapenemase genes was also performed (Table S3). Thirty-one isolates had the same species identification as MALDI-TOF MS, and four isolates showed a discrepancy between MALDI-TOF MS and 16S rRNA sequencing. Three isolates of *A. caviae* were identified as *A. hydrophila* by 16S sequencing, and *A. salmonicida* was identified as *A. media* by 16S sequencing.

**Figure 2:**
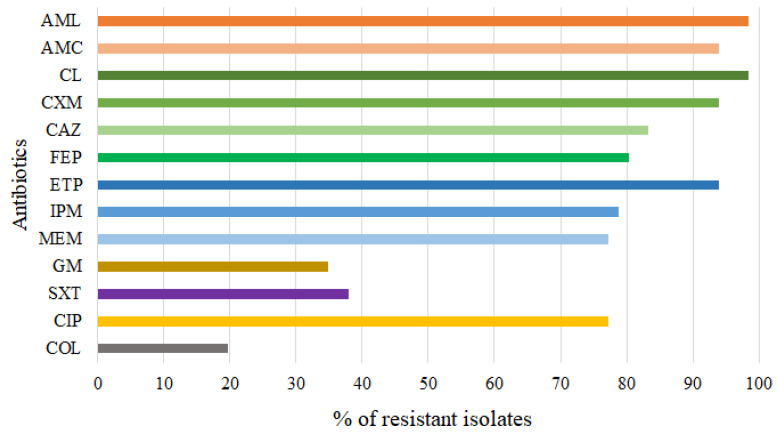
Antibiotic resistance patterns of 66 *Aeromonas* isolates. AML = amoxicillin, AMC = amoxicillin/clavulanic acid, CL = cephalexin, CXM = cefuroxime, CAZ = ceftazidime, FEP = cefepime, ETP = ertapenem, IPM = imipenem, MEM = meropenem GM = gentamicin, SXT = trimethoprim/sulfamethoxazole, CIP = ciprofloxacin, COL = colistin.

All *Aeromonas* isolates were further subjected to antibiotic susceptibility testing against a range of clinically relevant antibiotics. More than 93% of the isolates were resistant to penicillins (amoxicillin (AML) and amoxicillin/clavulanic acid (AMC)) and first and second generation cephalosporins (cephalexin (CL) and cefuroxime (CXM)) (Figure 2). In addition, more than 80% of the isolates were resistant to third and fourth generation cephalosporins (ceftazidime (CAZ) and cefepime (FEP)) and carbapenems (ertapenem (ETP), imipenem (IPM), and meropenem (MEM)), and more than 77% were resistant to ciprofloxacin (CIP). On the other hand, the isolates studied showed lower resistance to gentamicin (GM) (35%), sulfamethoxazole–trimethoprim (SXT) (38%), and colistin (COL) (20%) (Figure 2).

Different levels of resistance to carbapenem antibiotics were found in different *Aeromonas* spp. Almost all *A. caviae*, *A. hydrophila*, *A. media*, and *A. veroni* were resistant to ertapenem (Table S1). In addition, almost all *A. media* and *A. veroni* were also resistant to imipenem and meropenem. In contrast, the prevalence of resistance of *A. caviae* and *A. hydrophila* to imipenem and meropenem averaged 75% and 82%, respectively (Table S1). Colistin resistance was detected in single isolates of *A. veronii* and *A. eucrenophila*, three isolates of *A. hydrophila* and eight isolates of *A. caviae* (Table S1). With the exception of *A. eucrenophila*, all of these colistin-resistant isolates were co-resistant to at least one of the three carbapenem antibiotics.

**Figure 3:**
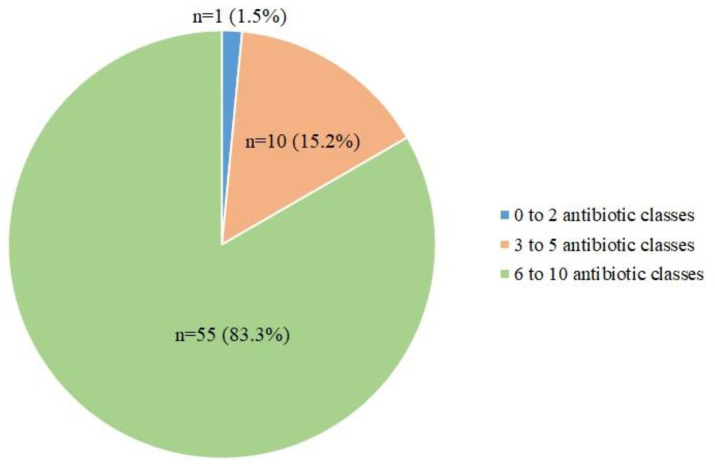
A pie chart showing the distribution of *Aeromonas* isolates resistant to a different number of antibiotic classes.

Almost all *Aeromonas* isolates (65/66) were multidrug-resistant (Figure 3), with 10 isolates showing co-resistance to 3–5 antibiotic classes and 55 isolates (majority *A. caviae*) showing co-resistance to 6–10 antibiotic classes (Figure 3).

We also phenotypically screened 15 *Aeromonas* isolates resistant to cefotaxime (3GC-resistant) for the production of ESBLs and plasmid-mediated AmpC (pAmpC) using the double-disc synergy test (DDST) and the combined disc test with phenylboronic acid (pAmpC test). The ESBL test identified all 3GC-resistant isolates (*n* = 15) as ESBL-positive, and the pAmpC test identified 11/15 positive isolates. We also tested all isolates (*n* = 66) for carbapenemase production using the Carba-NP test and identified 45/66 isolates as phenotypic carbapenemase producers.

### 2.2. Detection of ESBL, AmpC and Carbapenemase Genes

Polymerase chain reaction (PCR) of genomic DNA for the five clinically relevant carbapenemase genes (*bla*_KPC_, *bla*_NDM_, *bla*_IMP_, *bla*_VIM_, and *bla*_OXA-48_) and Sanger sequencing of the obtained amplicons were performed for all 66 *Aeromonas* isolates (Table 1). The most frequently detected carbapenemase gene was the *bla*_KPC-2_ gene, which was found in 41 isolates (62%), 6 of which co-occurred with *bla*_VIM-2_ (in *A. caviae*), and 1 with *bla*_IMP-13_ (in *A. media*). Two *A. caviae* isolates were positive for the *bla*_NDM-1_ gene and one for the *bla*_OXA-48_ gene. Isolates identified as ESBL producers (*n* = 15) were screened for ESBL genes (*bla*_CTX-M_ groups 1, 2, 9, *bla*_TEM_, *bla*_SHV_, *bla*_PER_, *bla*_VEB_, *bla*_GES_ and *bla*_SME_) by PCR and Sanger sequencing of the obtained amplicons (Table 1). Ten isolates were shown to carry at least one of the ESBL genes targeted. The most frequently detected ESBL gene was *bla*_GES-5_, which was found in 8 of 10 isolates (in 5 *A. caviae* and 3 *A. media*). In two isolates (*A. caviae* and *A. media*), *bla*_GES-5_ co-occurred with *bla*_TEM-1_ and in one isolate with *bla*_SHV-12_ (in *A. media*). One *A. caviae* isolate carried *bla*_GES-5+TEM-1+CTX-M-15_ and one carried *bla*_GES-5+TEM-1+OXA-48_. The gene *bla*_VEB-9_ was detected in one *A. hydrophila* isolate (Table 1). Of the 11 strains that produced pAmpC, 6 carried *bla*_MOX_ (5 *A. caviae*, 1 *A. hydrophila*), 1 of them together with *bla*_CIT_ (*A. caviae*), 1 with *bla*_FOX_ (*A. caviae*), and 1 with both *bla*_CIT_ and *bla*_FOX_ (*A. caviae*). Two isolates carried only *bla*_CIT_, one carried only *bla*_FOX_, and two contained *bla*_ACC_. None of the *mcr* genes were detected in the colistin-resistant strains (*n* = 13).

To determine whether the ESBL and carbapenemase genes were placed on plasmids, plasmid DNA was subjected to target PCR for the corresponding genes. Out of 15 ESBL-producing isolates, 2 *A. media* isolates carried *bla*_GES-5_ and 1 carried *bla*_SHV-12_ on plasmids. The *bla*_CTX-M-15_ gene was found on plasmid DNA from one *A. caviae*. All 5 isolates carrying *bla*_TEM-1_ were confirmed to carry it on plasmids. Regarding the carbapenemase genes, 40/41 isolates carried *bla*_KPC-2_ on plasmids. The gene *bla*_VIM-2_ was found on plasmids of 6/7 *A. caviae* isolates, and 1 *A. caviae* carried *bla*_NDM-1_ on the plasmid. The *bla*_OXA-48_ and *bla*_IMP-13_ genes were not detected on the plasmids.

Isolates that had ESBL and/or carbapenemase genes on a plasmid (*n* = 47) were analyzed for the presence of plasmid replicon types by PCR-based replicon typing (PBRT) (Table 1). No plasmid replicons were detected in 4 *Aeromonas* isolates that were positive for carbapenemase genes by regular PCR (Table 1). In the remaining 43 isolates, 17 different plasmid replicon types were detected, with 10 isolates having one plasmid type and 33 isolates having more than one plasmid. The most common plasmid replicon type detected in *Aeromonas* isolates was ColE (*n* = 38). The combination of two plasmid replicon types (ColE + IncR, ColE + IncK, ColE + IncU, ColE + IncW, ColE + IncFIC, and IncW + IncP) was detected in 18 isolates, while the combination of three (ColE + IncR + IncHI1, ColE + IncR + IncP, ColE + IncN + IncY, ColE + IncHI1 + IncN, ColE + IncY + FrepB, and ColE + IncR + IncU) and four replicon types (ColE + IncU + IncK + IncFrepB, and IncR + IncU + IncL/M + IncFrepB) were detected in 9 and 4 isolates, respectively. Two *A. caviae* isolates had six plasmids (ColE + IncU + IncHI1 + IncL/M + IncFrepB + IncK, and ColE + IncI1-1y + IncFIA + IncW + IncY + IncFrepB) (Table 1).

### 2.3. Enterobacterial Repetitive Intergenic Consensus Polymerase Chain Reaction (ERIC-PCR) Fingerprinting

Genetic similarity of all 66 *Aeromonas* spp. was assessed by ERIC-PCR analysis (Figure S1A–D). The fingerprints of the isolates consisted of 1 to 13 amplification bands ranging in size from 100 bp to 10,000 bp. Overall, the examined isolates exhibited great intraspecies diversity as they were assigned to 50 different ERIC-PCR profiles (27/38 for *A. caviae*, 9/11 for *A. hydrophila*, 7/7 for *A. media*, and 7/7 for *A. veronii*).

### 2.4. Conjugal Transfer of Carbapenem Resistance and Genotypic Characterization of Captured Plasmids

Conjugation experiments were performed to determine the possible transfer of carbapenem resistance from *Aeromonas* spp. to other bacteria. These experiments were performed using 10 randomly selected carbapenem-resistant *Aeromonas* isolates carrying carbapenemase genes on a plasmid (4 *A. caviae*, 2 *A. hydrophila*, 1 *A. media*, 2 *A. veronii*, and 1 *A. salmonicida*) as donors and the susceptible *E. coli* CV601 as the recipient. On LB plates containing meropenem (0.12 mg/L), we selected transconjugants that matched the phenotype of the donors. Transfer of the meropenem resistance phenotype was successful in three of ten isolates tested, namely, one KPC-2-carrying *A. caviae*, one NDM-1-carrying *A. caviae*, and one KPC-2-carrying *A. salmonicida* (Table 1). The transfer frequency of meropenem-resistant transconjugants was 6.41 × 10^−8^ and 7.52 × 10^−5^ per recipient in KPC-2-carrying *A. caviae* and *A. salmonicida*, respectively, and 8.59 × 10^−7^ per recipient in NDM-1-carrying *A. caviae.*

Plasmid DNA was extracted from all three transconjugants and analysed by PCR for the presence of carbapenemase genes and plasmid replicon types (Table 1). The carbapenemase gene *bla*_KPC-2_ was detected in transconjugants from *bla*_KPC-2_-PCR positive *A. caviae* and *A. salmonicida*, whereas *bla*_NDM-1_ was not detected in transconjugant from *bla*_NDM-1_-PCR positive *A. caviae*. In transconjugants from *bla*_NDM-1_-PCR positive *A. caviae* and *bla*_KPC-2_-PCR positive *A. salmonicida*, a plasmid was detected that was assigned to the ColE replicon type. In contrast, a combination of the replicon types of ColE and IncN plasmids was detected in transconjugant from *bla*_KPC-2_-PCR positive *A. caviae* (Table 1).

## 3. Discussion

The present study aimed to characterize *Aeromonas* spp. from treated municipal and untreated hospital wastewater that exhibited resistance to the antibiotics 3GCs and carbapenems, which are important for human health, using culture-dependent and culture-independent approaches. *Aeromonas* spp. are ubiquitous bacteria found in various aquatic habitats worldwide [9,11]. Considering this and the fact that they can easily acquire and exchange ARGs [30], research needs to focus more on the impact of these potentially harmful bacteria on human health.

In this study, 66 resistant *Aeromonas* strains were successfully isolated and identified to the species level by MALDI-TOF MS and/or 16S rRNA gene sequencing. These isolates could be assigned to 7 different species, with *A. caviae* (58%) dominating, followed by *A. hydrophila*, (17%), *A. media* (11%) and *A. veronii* (11%), while the prevalence of *A. eucrenophila*, *A. rivipollensis* and *A. salmonicida* was 2%. Of note, MALDI-TOF MS showed very good correlation with the results of 16S rRNA sequencing, with the exception of three cases of *A. caviae* identified as *A. hydrophila* and one case of *A. salmonicida* identified as *A. media* by 16S rRNA sequencing. In a previous study [31], MALDI-TOF MS was shown to be a more accurate method than 16S sequencing for distinguishing *Aeromonas* spp., particularly *A. caviae* and *A. hydrophila* and, therefore, we relied on the results from MALDI-TOF MS as the correct identification.

In other studies on aeromonads from wastewater environments, *A. caviae* was identified as the most abundant species in Brazil [32], while *A. veronii* was the predominant species in wastewater in Portugal [5]. Further assessment of genetic relatedness within these species by ERIC-PCR revealed high intraspecies diversity, as 7 species were assigned to 50 different ERIC-PCR profiles. This is in agreement with other studies that also showed high heterogeneity in ERIC sequences of *Aeromonas* strains [33,34,35,36].

Although *Aeromonas* spp. are environmental bacteria found primarily in aquatic environments, some members of this genus are pathogenic [13]. These include *A. caviae*, *A. dhakensis*, *A. hydrophila*, and *A. veronii*, which have been associated with gastrointestinal and respiratory tract infections, soft tissue and skin infections, etc. [11,12,13]. These species have also been isolated from untreated and treated wastewater [5,32]. Therefore, the predominant isolation of these species from hospital and municipal wastewater in this study suggests that these sources serve as reservoirs for these pathogens and as potential pathways for their transmission to humans and animals.

Characterizing the AR profile of *Aeromonas* isolates from wastewater is critical to understanding AR from the perspective of the One Health approach. In our study, all but one isolate was multidrug-resistant (i.e., resistant to ≥3 antibiotic classes). As expected, of all antibiotics tested, resistance to cephalosporins (first–fourth generation) and carbapenems (80%) was most common, followed by fluoroquinolones (CIP) (77%). Interestingly, 18% of our *Aeromonas* isolates (mainly *A. caviae* and *A. hydrophila*) were resistant to both carbapenems and colistin, limiting treatment options for *Aeromonas* infections, as carbapenems and colistin play an important role as “last resort” in the treatment of infections caused by gram-negative MDR bacteria. Therefore, the discharge of untreated hospital wastewater and treated municipal wastewater could increase the prevalence of these opportunistic pathogens with clinically relevant AR phenotypes in environmental waters and pose a threat to human health.

The positive AmpC β-lactamase result in some *Aeromonas* isolates was not unexpected as *Aeromonas* spp. are known to harbor chromosomal AmpC β-lactamase genes [11]. Among these genes, *bla*_MOX_ was the most prevalent. Previously, *bla*_MOX_ had been described in *A. caviae* [27] and in *Aeromonas* sp. from treated wastewater [14]. In addition, *Aeromonas sanarellii*, *A. caviae*, and *A. media* have been identified as carriers of three different variants of *bla*_MOX_ [37]. Many previous studies have shown that *Aeromonas* isolates from the environment were susceptible to the 3GCs [5,38,39]. However, in this study, 15 *Aeromonas* isolates were isolated on selective agar with cefotaxime (3GC), all of which were resistant to ceftazidime (3GC) and produced ESBLs. Four different ESBL genes (*bla*_GES-5_, *bla*_SHV-12_, *bla*_CTX-M-15,_ and *bla*_VEB-9_) and one narrow spectrum β-lactamase gene (*bla*_TEM-1_) were found in these isolates, with *bla*_GES-5_ being the predominant gene and found mainly in *A. caviae* and *A. media* isolates. This is consistent with a previous study in which the *bla*_GES-5_ gene was also found in *A. caviae* isolated from hospital wastewater in Brazil [29]. Although this gene is mainly of chromosomal origin in *A. caviae* and *A. media* isolates, it was also detected in an *A. media* isolate on the plasmid replicon ColE which, to our knowledge, had not previously been associated with the carriage of the *bla*_GES-5_ gene. The other ESBL genes detected have also been found in environmental *Aeromonas* spp. in previous studies [10,28,40,41].

The majority of *A. caviae* (37/38), *A. hydrophila* (7/8), and *A. media* (6/7) that were resistant to at least one of the carbapenem antibiotics tested produced carbapenemases and mainly possessed the *bla*_KPC-2_ gene. Previous studies have demonstrated the presence of *bla*_KPC-2_ in *Aeromonas* strains from natural waters in Brazil [42,43] and from wastewater in Brazil [3], the United States [30], China [20], and Japan [18]. Besides the present study, only one previous study detected *bla*_KPC-2_ in *Aeromonas* sp. from activated sludge in Europe (Poland) [14]. In addition, in the present study, *bla*_VIM-2_, *bla*_NDM-1,_ and *bla*_OXA-48_ were detected in *A. caviae*, while *bla*_IMP-13_ was found in *A. media*. To our knowledge, the *bla*_VIM-2_, *bla*_OXA-48_, and *bla*_IMP-13_ genes have never been detected in *Aeromonas* strains, and the *bla*_NDM-1_ gene was only recently discovered in two separate clinical cases of *A. caviae* in China [24,25]. Some of these genes (e.g., *bla*_OXA-48_, *bla*_IMP-13_) in the *Aeromonas* isolates studied in this work were mainly of chromosomal origin. However, most of our *Aeromonas* isolates carried carbapenemase genes on plasmids, especially *bla*_KPC-2_, *bla*_VIM-2_, and *bla*_NDM-1_, suggesting that they were acquired by HGT, probably under the selection pressure of antibiotics in hospital and municipal wastewater [2]. Interestingly, in six *A. caviae* isolates *bla*_KPC-2_ co-occurred with *bla*_VIM-2_ on a plasmid DNA, while in one *A. media bla*_KPC-2_ co-occurred with *bla*_IMP-13_ but was not present on plasmids. To our knowledge, this is the first report of the co-occurrence of both gene combinations (*bla*_KPC-2_ + *bla*_VIM-2_ and *bla*_KPC-2_ + *bla*_IMP-13_) in *Aeromonas* strains.

Plasmid-mediated multidrug resistance plays an important role in the worldwide spread of ARGs [44]. This study showed that *Aeromonas* isolates harbored various plasmid replicons, with ColE (*n* = 38), IncR (*n* = 15) and IncU (*n* = 11) being the most frequently detected. Our carbapenem-resistant *Aeromonas* isolates carrying these plasmids were also positive for *bla*_KPC-2_, *bla*_VIM-2_, or *bla*_NDM-1_ genes, suggesting possible mobilization of these genes by plasmid-mediated transfer. The plasmid replicons ColE and IncR have been associated with the carriage of *bla*_KPC-2_ in *Enterobacterales* isolates [45,46,47] while IncU (also known as IncP-6 [48]) has been associated with the transfer of *bla*_KPC-2_ from *Aeromonas* to *E. coli* [18]. In addition to *bla*_KPC-2_, other carbapenamase genes such as *bla*_NDM-1_ and *bla*_OXA-48_ [49,50] and ESBL genes such as *bla*_CTX-M-15_ [51] have also been found on the ColE plasmid in enterobacteria. To our knowledge, this is one of the first reports of *Aeromonas* isolates carrying *bla*_KPC-2_, *bla*_VIM-2_ or *bla*_NDM-1_ genes associated with conjugative ColE plasmids.

In the present study, transferable carbapenem (meropenem)-resistant plasmids were also captured from two *A. caviae* and one *A. salmonicida* strains to carbapenem-susceptible *E. coli* CV601. One ColE plasmid was captured from *A. caviae* (*bla*_NDM-1_ positive) and *A. salmonicida* (*bla*_KPC-2_ positive), and another one was captured from *A. caviae* (*bla*_KPC-2_ positive) in combination with replicon type IncN. The presence of *bla*_KPC-2_ in the ColE plasmid in transconjugant from *A. salmonicida* suggests that these plasmids may play an important role in the spread of *Aeromonas* carrying plasmid-localized *bla*_KPC-2_ genes. Moreover, the observation made in this study that two plasmids (ColE and IncN) were co-transferred from *A. caviae* (*bla*_KPC-2_ positive) to *E. coli* is also interesting in light of the findings of Barry et al. (2019) [52]. They demonstrated co-transfer of small cryptic plasmids such as Col440I along with larger plasmids carrying *bla*_KPC-2_ from *bla*_KPC_-positive *Citrobacter freundii* to *E. coli*. They found that the larger *bla*_KPC_-plasmids were never transferred alone and, therefore, hypothesized that these small plasmids are often transferred with conjugative plasmids carrying ARGs because they might play a helper role. The same could be true for our small (ColE) and larger (IncN) plasmids detected in *bla*_KPC_-positive transconjugant. The conjugative plasmid replicon IncN has already been associated with the carriage of *bla*_KPC-2_ in enterobacteria [53]. However, we cannot exclude the possibility that *bla*_KPC-2_ is present on both plasmids or only on ColE. In addition, *bla*_NDM-1_ was not detected in the transconjugant from NDM-1-positive *A. caviae*, suggesting that it may be localized on other mobile genetic element that is integrated into the host chromosome. 

*Aeromonas* spp. are considered susceptible to colistin, with the exception of *Aeromonas jandaei* and *A. hydrophila* [54,55]. In our study, 3/11 *A. hydrophila* were resistant to colistin, one *A. veronii* and one *A. eucrenophila*, and most *A. caviae* (8/38). Although *mcr* genes (*mcr*-1, *mcr*-3, and *mcr*-5) have been found previously in *Aeromonas* spp. [56,57,58], our *A. caviae*, *A. eucrenophila* and *A. hydrophila* isolates were negative for all *mcr* genes tested (*mcr*1–5). Nevertheless, it is possible that these isolates have a novel variant of the *mcr* gene or another resistance mechanism, such as the addition of L-Ara-4N to the lipopolysaccharide layer [59]. Therefore, whole genome sequencing of these isolates would be required to fully elucidate the mechanism of colistin resistance.

## 4. Materials and Methods

### 4.1. Wastewater Sampling

Treated municipal wastewater (secondary effluent; 24-h composite samples) and untreated hospital wastewater (grab samples) from two hospitals (H1 and H2) in Zagreb, Croatia were collected on three consecutive days in winter 2020 (January). Municipal wastewater underwent stepwise treatment (primary and secondary-aerobic biodegradation) prior to discharge into the Sava River. Hospital wastewater was not treated in the hospital prior to discharge into the municipal wastewater system, as is common practice in all hospitals in Croatia. Samples were collected in sterile glass bottles (2.5 L), transported in cool boxes with ice blocks, and processed in the laboratory within 2 h.

### 4.2. Isolation of 3GC- and Carbapenem-Resistant Aeromonas

A series of dilutions of municipal and hospital wastewater samples were prepared in 0.85% NaCl (tenfold dilution up to 1:10,000). The dilutions were filtered in triplicate under vacuum through sterile mixed cellulose ester membrane filters (47 mm diameter, 0.22 µm pore size, Whatman, GE Healthcare, Life Science, Chicago, IL, USA) and the filters were placed on Rapid’ *E. coli* 2 (BioRad, Hercules, CA, USA) agar plates supplemented with 4 mg/L cefotaxime (CTX) and CHROMagar mSuperCARBA (CHROMagar, Paris, France) agar plates. The plates were incubated at 37 °C for 24 h. Different colonies of Rapid’ *E. coli* 2 + CTX and CHROMagar mSuperCARBA were selected for isolation of presumptive 3GC- or carbapenem-resistant *Aeromonas* spp. A total of 200 presumptive isolates were purified on the same medium and stored in a 20% glycerol stock at −80 °C.

### 4.3. Identification of Isolates

Isolates were identified to species level by MALDI-TOF MS. Isolates were streaked on Mueller–Hinton plates (Oxoid, Basingstoke, UK), incubated overnight for 18–24 h at 37 °C, and sent to the Laboratory of Mass Spectrometry and Functional Proteomics at the Ruđer Bošković Institute. Pure cultures were transferred directly to the spots of the MALDI-TOF MS target using a toothpick. A score value greater than 2.00 for the species level and a score value between 1.70 and 1.99 was indicated for successful identification. Isolates that could not be identified to species level by MALDI-TOF MS and those that carried carbapenemase gene(s) were additionally analyzed by sequencing of the 16S rRNA gene. The 1465 pb fragment was amplified with the universal primers 27F (5′-AGAGTTTGATCCTGGCTCAG-3′) and 1492R (5′-GGTTACCTTGTTACGACTT-3′) [60]. Thermocycling conditions were as follows: initial denaturation at 98 °C for 5 min, followed by 35 amplification cycles of denaturation for 10 s at 98 °C, annealing for 30 s at 60 °C and extension for 1:30 min at 72 °C, followed by a final extension at 72 °C for 5 min. PCR products were sent to Macrogen Europe (Amsterdam, The Netherlands) for purification and Sanger sequencing. Partial nucleotide sequences of the 16S rRNA genes were compared with the homologous sequences of the different *Aeromonas* species available in the GenBank database using *BLAST* (https://blast.ncbi.nlm.nih.gov/Blast.cgi, accessed on 20 February 2023). All sequences were identified to species level (≥99% sequence identity).

### 4.4. Antibiotic Susceptibility Testing and Phenotypic Identification of ESBLs, pAmpC and Carbapenemases

Antibiotic susceptibility testing was performed by the disk diffusion method using commercially available disks according to EUCAST guidelines [61]. Antibiotics tested included AML (25 µg), AMC (30 µg, BD BBL), CL (30 µg, OXOID), CXM (30 µg, BD BBL), CAZ (10 µg, OXOID), FEP (30 µg, BD BBL), ETP (10 µg, BD BBL), IPM (10 µg, BD BBL), MEM (10 µg, BD BBL), GM (10 µg, BD BBL), SXT (1.25/23.75 µg, BD BBL), and CIP (5 µg, BD BBL). Antibiotic disks AML, CL, and CAZ were purchased from OXOID (Basingstoke, UK) and the others from BD BBL (Franklin Lakes, NJ, USA). In addition, for isolates resistant to any of the carbapenems, the minimum inhibitory concentration (MIC) was determined by broth microdilution according to EUCAST guidelines. In addition, the susceptibility to COL was tested for all isolates using the MIC. Briefly, the initial concentration of 64 mg/L (COL, IPM, and MEM) or 16 mg/L (ETP) was serially double diluted in a sterile 96-well plate to a final concentration of 1 mg/L (COL, IPM, MEM) or 0.25 mg/L (ETP). Wells contained 90 μL Mueller–Hinton broth (Merck, Darmstadt, Germany) or, in the case of colistin, cation-adjusted Mueller–Hinton broth 2 (Sigma-Aldrich, Steinheim am Albuch, Germany) and serially diluted antibiotics. Each well was inoculated with 10 μL of an overnight bacterial culture diluted to a concentration of 5 × 10^5^ CFU/mL. The plates were incubated overnight at 37 °C, and the lowest concentration at which no visible growth occurred was determined as the MIC of the isolates. *Escherichia coli* ATCC 25,922 and *Escherichia coli* NCTC 13,846 were used for quality control.

For phenotypic determination of ESBL and pAmpC production, 3GC-resistant isolates were subjected to DDST and pAmpC tests, respectively. For ESBL production, overnight cultures were diluted in 0.85% NaCl to a concentration of 0.5 McFarland and plated onto Mueller–Hinton agar using a sterile cotton swab. The CAZ (30 µg) and CTX (30 µg) discs were placed 20 mm and 30 mm (centre to centre) away from the amoxicillin-clavulanate (AMC, 20 + 10 µg) disc, respectively. If, after overnight incubation at 37 °C, synergy with clavulanate occurred with one of the 3GCs (enlargement of the zone of inhibition), this was considered a positive result for ESBL production (EUCAST guidelines).

The pAmpC production was determined using a cefoxitin disk (30 µg) alone and in combination with phenylboronic acid (300 µg) [62] applied to the inoculated Muller–Hinton plates. If there was an increase in the zone of inhibition of ≥5 mm after overnight incubation at 37 °C, the isolate was classified as a pAmpC producer.

All isolates that were resistant to carbapenems (both 3GC- and carbapenem-resistant) were subjected to an in-house CarbaNP assay to determine carbapenemase production [63]. Briefly, bacterial suspensions in Tris-HCL lysis buffer were mixed with 100 µL phenol red solution containing ZnSO_4_ × 7H_2_O (0.1 mM) and imipenem-cilastatin (12 mg/mL). After incubation at 37 °C for a maximum of 2 h, the bacterial strains that changed the color of the suspension from red to orange or yellow were classified as carbapenemase producers.

### 4.5. Genomic and Plasmid DNA Extraction

Genomic DNA was extracted from overnight cultures of all *Aeromonas* isolates and transconjugants using the Quick-DNA^TM^ Miniprep Plus Kit (Zymo, Irvine, CA, USA) according to the manufacturer’s instructions. Plasmid DNA was extracted from overnight cultures of ESBL-, pAmpC-, and carbapenemase-producing *Aeromonas* isolates and three carbapenem-resistant transconjugants using the ExtractNow Plasmid Mini Kit (Minerva Biolabs, Berlin, Germany) according to the manufacturer’s protocol. The extracted genomic and plasmid DNA was stored at −20 °C until further analysis.

### 4.6. Detection of Target ARGs by PCR and Sanger Sequencing of the Amplicons

Target ARGs in genomic and plasmid DNA extracted from *Aeromonas* isolates and transconjugants were detected by regular PCR with specific primers and conditions (Table 2), and the gene variant was identified by Sanger sequencing of the amplicons obtained. 3GC-resistant isolates (*n* = 15) were screened for ESBL genes by singleplex PCR (*bla*_CTX-M_ groups 1, 2, and 9) and multiplex PCR (*bla*_TEM_, *bla*_SHV_, *bla*_PER_, *bla*_VEB_, *bla*_GES,_ and *bla*_SME_) and for pAmpC genes (*bla*_FOX_, *bla*_EBC_, *bla*_CIT_, *bla*_ACC_, *bla*_MOX_) by multiplex PCR (Table 2). All *Aeromonas* isolates (*n* = 66) and three transconjugants were screened for the presence of carbapenemase genes (*bla*_KPC_, *bla*_NDM_, *bla*_OXA-48_-like, *bla*_IMP_, and *bla*_VIM_). The colistin-resistant strains were screened for the *mcr*-1, *mcr*-2, *mcr*-3, *mcr*-4, and *mcr*-5 genes (Table 2). All PCR products were separated by 1.5% gel electrophoresis at 100 V for 60 min, stained with ethidium bromide, and visualized in the UV transilluminator. All positive PCR products were sent to Macrogen (Amsterdam, The Netherlands) for purification and sequencing in forward direction. The resulting sequences were compared with the reference in the NCBI database using BLASTX search.

### 4.7. Plasmid Replicon Typing

PBRT was used to identify the replicon type of plasmids in *Aeromonas* isolates and transconjugants. This was achieved by PCR amplification with plasmid DNA of the strains and transconjugants using the specific primer sets for 22 replicons and conditions listed in Table S2 [64,65,66]. Reactions were performed using the Hot Start Core Kit Ab+ (Jena Bioscience, Jena, Germany). PCR products were separated by electrophoresis (100 V, 60 min) on a 1.5% agarose gel and stained with ethidium bromide.

**Table 2:** Specific primers and conditions for polymerase chain reaction (PCR) of resistance genes.

Target Gene		Primer Name	Primer Nucleotide Sequence (5′–3′)	Amplicon Size (bp)	PCR Conditions	Reference
Carbapenemases	*bla*_KPC_	KPC-F	AGTTCTGCTGTCTTGTCT	793	Initial denaturation step at 94 °C for 2:30 min; 30 cycles of 94 °C for 20 s, annealing temperature: 57 °C for *bla_KPC_*, *bla_VIM_*, *bla_OXA-48_*, 55 °C for *bla_IMP_*, 58 °C for *bla_NDM_* for 25 s; 72 °C for 45 s; final extension 72 °C for 2 min	[67]
		KPC-R	CTTGTCATCCTTGTTAGGC			
	*bla*_VIM_	VIM MJ-F	GGTGAGTATCCGACAGTC	442		
		VIM-MJ-R	CAGCACCRGGATAGAAGAG			
	*bla*_IMP_	IMP MJ-F1	GGYGTTTATGTTCATACWTC	235		
		IMP MJ-R1	GGATYGAGAATTAAGCCACTC			
	*bla*_OXA-48_	OXA-48A	TTGGTGGCATCGATTATCGG	744		[68]
		OXA-48B	GAGCACTTCTTTTGTGATGGC			
	*bla*_NDM_	NDM-F	TGGCAGCACACTTCCTATC	813		[69]
		NDM-R	AGATTGCCGAGCGACTTG			
*bla*_CTX-M_	*bla*_CTX-M-1_	M13U	GGTTAAAAAATCACTGCGTC	864	Initial denaturation step at 94 °C for 2:30 min; 30 cycles of 94 °C for 20 s, annealing temperature: 55 °C for 25 s; 72 °C for 45 s; final extension 72 °C for 2 min	[70]
		M13L	TTGGTGACGATTTTAGCCGC			
	*bla*_CTX-M-2_	M25U	ATGATGACTCAGAGCATTCG	866		
		M25L	TGGGTTACGATTTTCGCCGC			
	*bla*_CTX-M-9_	M9U	ATGGTGACAAAGAGAGTGCA	870		
		M9L	CCCTTCGGCGATGATTCTC			
ESBL-multiplex 1	*bla*_TEM_	TEM-F	GCGGTAAGATCCTTGAGAGT	620	Initial denaturation step at 94 °C for 2:30 min; 35 cycles of 94 °C for 30 s, annealing temperature: 55 °C for 30 s; 72 °C for 45 s; final extension 72 °C for 2 min	[67]
		TEM-R	TACGATACGGGAGGGCTTA			
	*bla*_SHV_	SHV-F	TTCGCCTGTGTATTATCTCC	494		
		SHV-R	CGCCTCATTCAGTTCCG			
	*bla*_PER_	PER-F	CTGGGCTCCGATAATGA	349		
		PER-R	CTGGTCGCCWATGATGA			
ESBL-multiplex 2	*bla*_VEB_	VEB-F	ATGCCAGAATAGGAGTAGC	673	Initial denaturation step at 94 °C for 2:30 min; 35 cycles of 94 °C for 30 s, annealing temperature: 58 °C for 30 s; 72 °C for 45 s; final extension 72 °C for 2 min	
		VEB-R	AATTGTCCATTCGGTAAAGTAAT			
	*bla*_GES_	GES-F	CTAGCATCGGGACACAT	504		
		GES-R	GACAGAGGCAACTAATTCG			
	*bla*_SME_	SME-F	GCTCAGGTATGACATTAGGT	350		
		SME-R	CCAATCAGCAGGAACACTA			
AmpC- multiplex	*bla*_FOX_	FOX-F	AACATGGGGTATCAGGGAGATG	190	Initial denaturation step at 94 °C for 2:30 min; 35 cycles of 94 °C for 30 s, annealing temperature: 55 °C for 30 s; 72 °C for 45 s; final extension 72 °C for 2 min	[71]
		FOX-R	CAAAGCGCGTAACCGGATTGG			
	*bla*_EBC_	EBC-F	TCGGTAAAGCCGATGTTGCGG	302		
		EBC-R	CTTCCACTGCGGCTGCCAGTT			
	*bla*_CIT_	CIT-F	TGGCCAGAACTGACAGGCAAA	462		
		CIT-R	TTTCTCCTGAACGTGGCTGGC			
	*bla*_ACC_	ACC-F	AACAGCCTCAGCAGCCGGTTA	346		
		ACC-R	TTCGCCGCAATCATCCCTAGC			
	*bla*_DHA_	DHA-F	AACTTTCACAGGTGTGCTGGGT	405		
		DHA-R	CCGTACGCATACTGGCTTTGC			
	*bla*_MOX_	MOX-F	GCTGCTCAAGGAGCACAGGAT	520		
		MOX-R	CACATTGACATAGGTGTGGTGC			
*mcr*-multiplex	*mcr*-1	mcr-1-F	AGTCCGTTTGTTCTTGTGGC	320	Initial denaturation step at 94 °C for 15 min; 25 cycles of 94 °C for 30 s, annealing temperature: 58 °C for 1:30 min; 72 °C for 1 min; final extension 72 °C for 10 min	[72]
		mcr-1-R	AGATCCTTGGTCTCGGCTTG			
	*mcr*-2	mcr-2-F	CAAGTGTGTTGGTCGCAGTT	715		
		mcr-2-R	TCTAGCCCGACAAGCATACC			
	*mcr*-3	mcr-3-F	AAATAAAAATTGTTCCGCTTATG	929		
		mcr-3-R	AATGGAGATCCCCGTTTTT			
	*mcr*-4	mcr-4-F	TCACTTTCATCACTGCGTTG	1116		
		mcr-4-R	TTGGTCCATGACTACCAATG			
	*mcr*-5	mcr-5-F	ATGCGGTTGTCTGCATTTATC	1664		
		mcr-5-R	TCATTGTGGTTGTCCTTTTCTG			

### 4.8. ERIC-PCR

All *Aeromonas* were fingerprinted by ERIC-PCR using the primers ERIC-1R (5′-ATGTAAGCTCCTGGGGATTCAC-3′) and ERIC-2 (5′-AAGTAAGTGACTGGGGTGAGCG-3′) [73]. The temperature profiles for amplification were as follows: initial denaturation at 95 °C for 7 min, followed by 30 cycles of amplification with denaturation for 30 s at 90 °C, annealing for 1 min at 52 °C, and extension for 8 min at 65 °C, followed by a final extension at 65 °C for 16 min [33]. The amplification products were separated by 1.5% gel electrophoresis for 90 min at 100 V. The size of the amplified products was determined by comparison with a 1-kb DNA ladder (Promega, Fitchburg, WI, USA).

The pattern of DNA fingerprint bands generated by ERIC-PCR was analyzed, and dendrograms were generated with GelJ software v2.0 [74], using the Dice coefficient to calculate similarity between fingerprints and UPGMA (the unweighted pair-group method with average linkages) method for cluster analysis.

### 4.9. Conjugation Assay

To investigate the potential plasmid transfer of carbapenem resistance, in vitro conjugation experiments were performed using 10 different carbapenemase-producing *Aeromonas* strains (4 *A. caviae*, 2 *A. hydrophila*, 2 *A. veronii*, 1 *A. media*, 1 *A. salmonicida*) as donors and the rifampicin- and kanamycin-resistant *E. coli* strain CV601 as plasmid recipient. The filter conjugation assay was performed as previously described [75]. Briefly, donor and recipient strains were grown overnight at 28 °C in LB broth. A total of 500 μL of the overnight cultures of each donor and recipient strain were mixed, centrifuged, and the pellets were resuspended in 200 µL of physiological saline and then spotted onto a filter for mating. After overnight incubation at 28 °C, the filters were washed in physiological saline, and 100 μL of the conjugation mixture was spread on LB agar plates containing kanamycin (50 mg/L), rifampicin (50 mg/L), and MEM (0.12 mg/L) and incubated for 48 to 72 h at 28 °C. Transconjugants were determined by the green fluorescence of the green fluorescent protein. Putative transconjugants were verified by BOX-PCR as previously described [76] and tested for carbapenemase genes and plasmid types as described above. Transfer frequencies were calculated as the total number of transconjugants divided by the total number of recipients.

### 4.10. Data Accessibillity

The Sanger sequence data were submitted to NCBI GenBank, and the accession numbers for each gene are as follows: OQ348696-OQ348735 (*bla*_KPC_), OQ348736 (*bla*_OXA-48_), OQ348737 (*bla*_IMP_), OQ348738-OQ348739 (*bla*_NDM_), OQ348740 (*bla*_CTX-M_), OQ348741-OQ348745 (*bla*_TEM_), OQ348746-OQ348752 (*bla*_GES_), OQ348753-OQ348758 (*bla*_VIM_), OQ348759 (*bla*_SHV_), OQ348760 (*bla*_VEB_), and OQ532911-OQ532961 (16S rRNA gene, Table S3).

## 5. Conclusions

This study showed that hospital and municipal wastewater contains multidrug-resistant *Aeromonas* species, some of which are opportunistic pathogens of clinical importance. These *Aeromonas* isolates exhibited a diverse plasmidome and harbored ESBL and/or carbapenemase genes, which are frequently localized on plasmids. Successful plasmid-mediated transfer of the carbapenem resistance phenotype from *A. caviae* and *A. salmonicida* strains to susceptible *E. coli* recipients was also demonstrated. All these data suggest that *Aeromonas* spp. in the environment may serve not only as a reservoir for clinically important carbapenemase genes but also as a source for their transfer to other bacteria, including pathogens.

## Figures and Tables

**Table 1:** Diversity, ESBL, pAmpC, and carbapenemase genes and incompatibility (Inc) groups of plasmids of resistant *Aeromonas* isolates and their respective transconjugants. Bold letters indicate isolates used for plasmid transfer. The asterisk * indicates isolate that successfully transferred carbapenemase resistance to the *E. coli* CV601 recipient. TWW = treated municipal wastewater, H = hospital wastewater.

Species	Isolates					Transconjugants	
	ID	Source	ARGs Detected on Genomic DNA	ARGs Detected on Plasmid DNA	Plasmid Replicons Detected	ARGs Detected on Plasmid DNA	Plasmid Replicons Detected
*A. caviae*	A36	TWW	KPC-2	KPC-2	ColE,		
	A1	TWW	KPC-2	KPC-2	ColE, R		
	A4	TWW	KPC-2	KPC-2	ColE, R		
	A9	TWW	KPC-2	KPC-2	ColE, R		
	A11	TWW	KPC-2	KPC-2	ColE, R		
	A23	TWW	KPC-2	KPC-2	ColE, R		
	**A3**	**TWW**	**KPC-2**	**KPC-2**	**ColE, R, P**		
	A20	TWV	KPC-2	KPC-2	ColE, R		
	A31	TWW	KPC-2	KPC-2	ColE, R, HI1		
	A6	TWW	KPC-2	KPC-2	ColE, U, HI1, L/M, FrepB, K		
	A19	TWW	KPC-2	KPC-2	ColE, U		
	A30	TWW	KPC-2	KPC-2	ColE		
	A2 *	**TWW**	**KPC-2**	**KPC-2**	**ColE, N, Y**	KPC-2	ColE, N
	A15	TWW	KPC-2	KPC-2	ColE, K		
	A32	TWW	KPC-2	KPC-2	ColE, K		
	A38	TWW	KPC-2	KPC-2	ColE, K		
	A8	TWW	KPC-2	KPC-2	K		
	A18	H	KPC-2	KPC-2	-		
	A21	TWW	KPC-2	KPC-2	P		
	A12	TWW	KPC-2, VIM-2	KPC-2, VIM-2	-		
	A35	TWW	KPC-2, VIM-2	KPC-2, VIM-2	ColE, R, HI1		
	A37	TWW	KPC-2, VIM-2	KPC-2, VIM-2	ColE, HI1, N		
	A29	TWW	KPC-2, VIM-2	KPC-2, VIM-2	ColE,		
	**A28**	**TWW**	**KPC-2, VIM-2**	**KPC-2, VIM-2**	**ColE, U**		
	A10	TWW	KPC-2, VIM-2	KPC-2, VIM-2	U		
	A24 *	**TWW**	**NDM-1**	**NDM-1**	**ColE**	-	ColE
	A14	TWW	CTX-M-15, TEM-1, GES-5, ACC	CTX-M-15, TEM-1	W, P		
	A22	TWW	TEM-1, GES-5, FOX, CIT, MOX	TEM-1, FOX, CIT, MOX	ColE, I1-1y, FIA, W, Y, FrepB		
	A26	TWW	TEM-1, MOX	TEM-1, MOX	ColE, W		
	A34	TWW	OXA-48; TEM-1, GES-5, MOX	TEM-1, MOX	ColE, Y, FrepB		
	A5	TWW	KPC-2	-	-		
	A13	TWW	VIM-2, ACC	-	-		
	A17	TWW	GES-5, CIT, MOX	CIT, MOX	-		
	A33	TWW	GES-5, MOX, FOX	MOX	-		
	A27	TWW	NDM-1, FOX	-	-		
	A7	TWW	-	-	-		
	A16	TWW	-	-	-		
	A25	TWW	-	-	-		
*A. hydrophila*	**A39**	**TWW**	**KPC-2**	**KPC-2**	**ColE, U, K, FrepB**		
	A40	TWW	KPC-2	KPC-2	ColE, U, K, FrepB		
	**A42**	**TWW**	**KPC-2**	**KPC-2**	**ColE, R**		
	A45	TWW	KPC-2	KPC-2	ColE, R		
	A48	TWW	KPC-2	KPC-2	ColE, U		
	A49	TWW	KPC-2	KPC-2	ColE		
	A41	TWW	KPC-2	KPC-2	-		
	A46	TWW	VEB-9	-	-		
	A47	TWW	MOX	-	-		
	A44	TWW,	-	-	-		
	A43	H	-	-	-		
*A. media*	A51	TWW	KPC-2	KPC-2	ColE,		
	A50	TWW	KPC-2	KPC-2	ColE, R, U		
	A56	TWW	KPC-2	KPC-2	ColE, R, U		
	**A55**	TWW	**KPC-2, IMP-13**	**KPC-2**	**ColE, R, U**		
	A54	TWW	KPC-2, GES-5	KPC-2, GES-5	-		
	A52	TWW	TEM-1, GES-5	TEM-1, GES-5	ColE,		
	A53	TWW	SHV-12, GES-5	SHV-12	ColE, FIC		
*A. veronii*	**A63**	**TWW**	**KPC-2**	**KPC-2**	**ColE, U, K, FrepB**		
	**A60**	**H**	**KPC-2**	**KPC-2**	**R, U, L/M, FrepB**		
	A62	H	-	-	-		
	A57	H	-	-	-		
	A58	H	-	-	-		
	A59	H	-	-	-		
	A61	TWW	-	-	-		
*A. salmonicida*	A64 *	**TWW**	**KPC-2**	**KPC-2**	**ColE, K**	KPC-2	ColE
*A. rivipollensis*	A65	TWW	CIT	-	-		
*A. eucrenophila*	A66	TWW	-	-	-		

## Data Availability

The data from this study have been deposited in NCBI GenBank, and the accession numbers for each gene are as follows: OQ348696–OQ348735 (*bla*_KPC_), OQ348736 (*bla*_OXA-48_), OQ348737 (*bla*_IMP_), OQ348738-OQ348739 (*bla*_NDM_), OQ348740 (*bla*_CTX-M_), OQ348741-OQ348745 (*bla*_TEM_), OQ348746-OQ348752 (*bla*_GES_), OQ348753-OQ348758 (*bla*_VIM_), OQ348759 (*bla*_SHV_), OQ348760 (*bla*_VEB_), and OQ532911-OQ532961 (16S rRNA gene).

## Supplementary Materials

The following supporting information can be downloaded at: https://www.mdpi.com/article/10.3390/antibiotics12030513/s1, Figure S1. Dendrogram showing genetic relatedness of (A) 38 *Aeromonas caviae*, (B) 11 *Aeromonas hydrophila*, (C) 7 *Aeromonas media* and (D) 7 *Aeromonas veronii* strains determined by analysis of ERIC-PCR fingerprints using the Dice similarity coefficient and UPGMA clustering method; Table S1: Antimicrobial susceptibility patterns of *Aeromonas* isolates; Table S2. Specific primers and conditions used in plasmid replicon typing; Table S3. Identification of *Aeromonas* strains by MALDI-TOF MS and/or 16S rRNA gene sequencing. Red letters indicate discrepancies between MALDI-TOF MS and 16S identification. ID represents the isolate designation; TWW and H refer to the source of bacterial isolation, treated municipal wastewater and hospital wastewater, respectively. ND, not determined.
