# Supplementary material for: Characterization of Third Generation Cephalosporin- and Carbapenem-Resistant Aeromonas Isolates from Municipal and Hospital Wastewater
Source: Antibiotics (Basel). 2023 Mar 3;12(3):513. doi: 10.3390/antibiotics12030513 (PMC10044312; doi:10.3390/antibiotics12030513)
Supplement: Supplementary file 1 [file antibiotics-12-00513-s001.zip › antibiotics-2221794-supplementary.pdf]

Supplementary Material for manuscript entitled

Characterization of third generation cephalosporin- and carbapenem-resistant *Aeromonas* isolates from municipal and hospital wastewater

*Sara Drk[a], Ana Puljko[a], Mia Dželalija[b], Nikolina Udiković-Kolić[a,] **

[a] Division for Marine and Environmental Research, Ruđer Bošković Institute, Bijenička 54, P.O. Box 180, 10 002 Zagreb, Croatia

[b]Department of Biology, Faculty of Science, University of Split, Ruđera Boškovića 33, 21 000 Split, Croatia

** Corresponding author: Dr. Nikolina Udiković-Kolić (nudikov@irb.hr), Division for Marine and Environmental Research, Ruđer Bošković Institute, Bijenička 54, P.O. Box 180; 10 002 Zagreb, Croatia, E-mail address: nudikov@irb.hr*

Figure S1. Dendrogram showing genetic relatedness of (A) 38 *Aeromonas caviae*, (B) 11 *Aeromonas hydrophila*, (C) 7 *Aeromonas media* and (D) 7 *Aeromonas veronii* strains determined by analysis of ERIC-PCR fingerprints using the Dice similarity coefficient and UPGMA clustering method.

A)

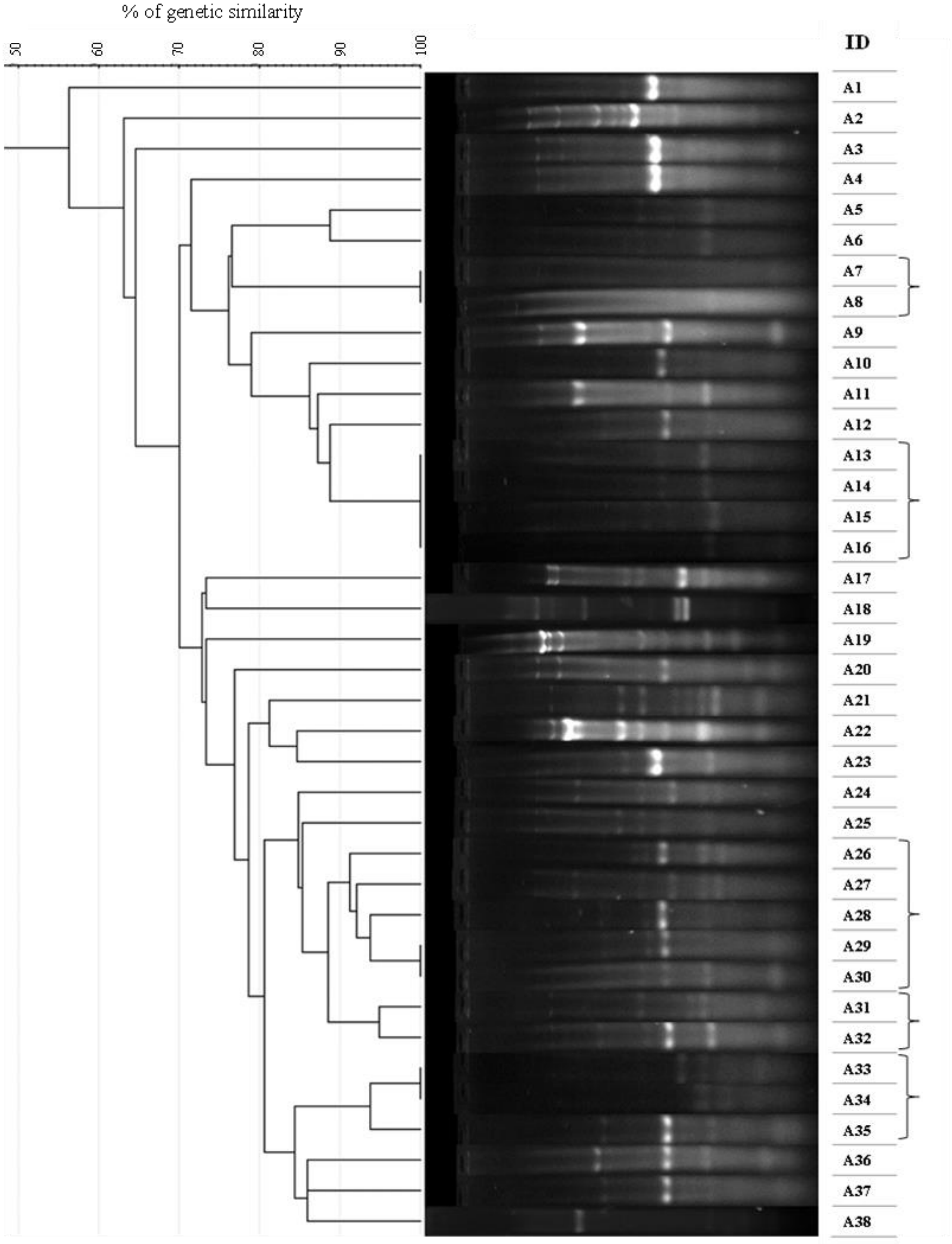

B)

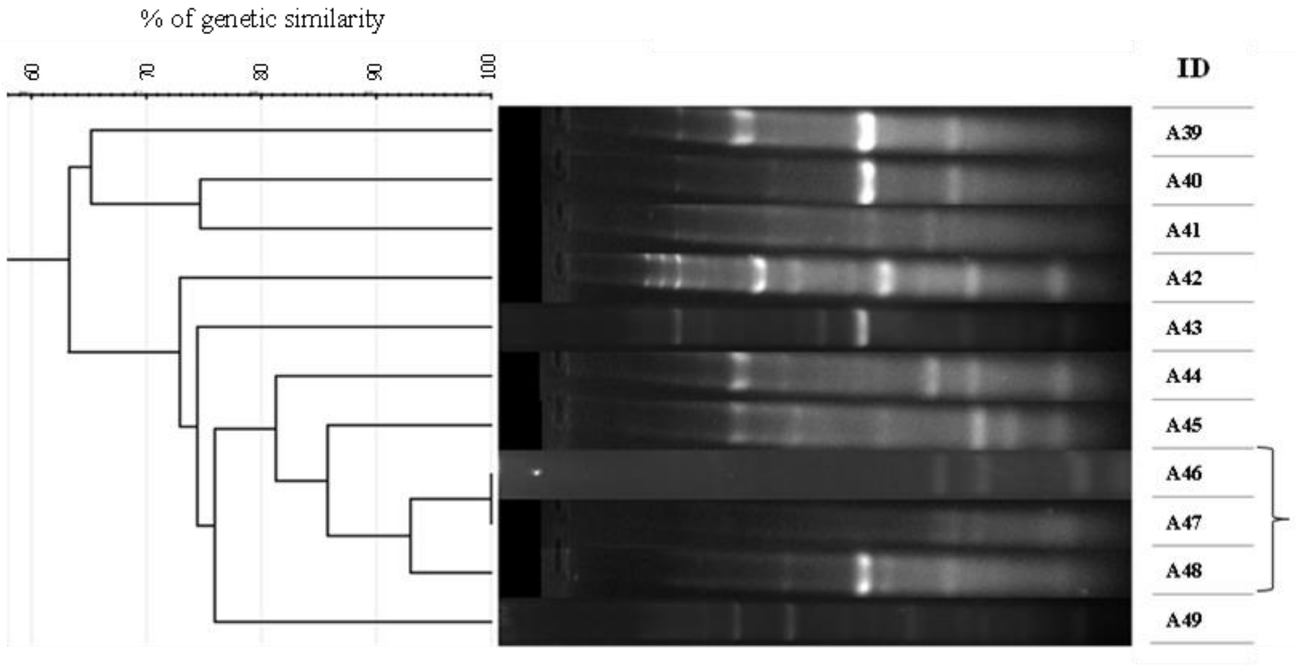
% of genetic similarity
60
70
80
90
100
ID
A39
A40
A41
A42
A43
A44
A45
A46
A47
A48
A49

C)

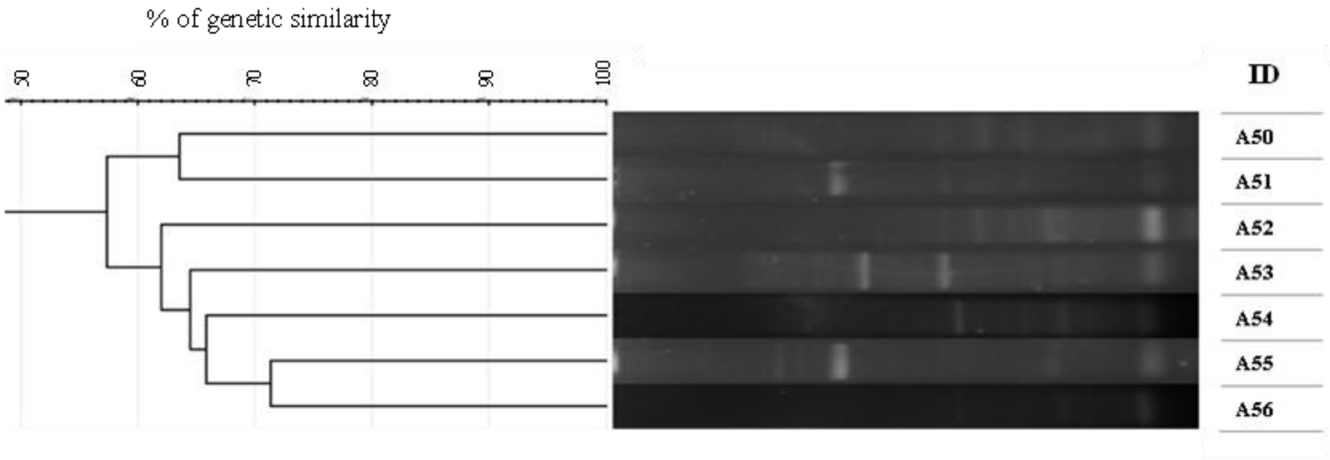
% of genetic similarity
50
60
70
80
90
100
ID
A50
A51
A52
A53
A54
A55
A56

D)

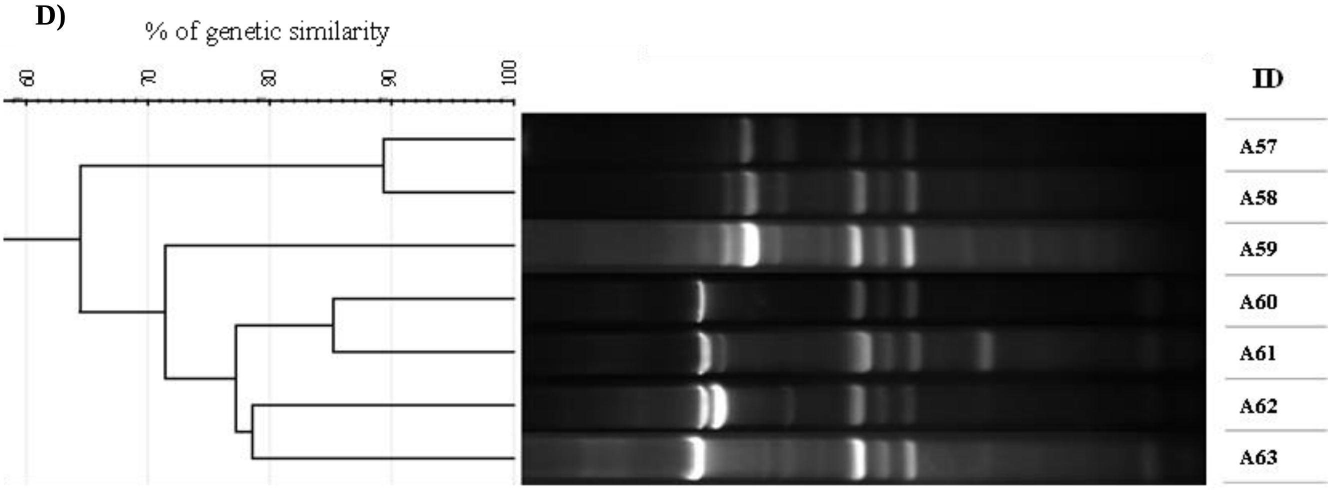
% of genetic similarity
60
70
80
90
100
ID
A57
A58
A59
A60
A61
A62
A63

Table S1. Antimicrobial susceptibility patterns of *Aeromonas* isolates. AML = amoxicillin, AMC = amoxicillin/clavulanic acid, CL = cephalexin, CXM = cefuroxime, CAZ = ceftazidime, FEP = cefepime, ETP = ertapenem, IPM = imipenem, MEM = meropenem GM = gentamicin, SXT = trimethoprim/sulfamethoxazole, CIP = ciprofloxacin, COL = colistin, S = susceptible, R = resistant

**Antibiotics**		***A. caviae***	***A. hydrophila***	***A. media***	***A. veronii***	***A. salmonicida***	***A. rivipollensis***	***A. eucrenophila***	**Total**
		No. of isolates (%)							
**AML**	S	0 (0)	0 (0)	0 (0)	1 (14)	0 (0)	0 (0)	0 (0)	1 (2)
	R	38 (100)	11 (100)	7 (100)	6 (86)	1 (100)	1 (100)	1 (100)	65 (98)
**AMC**	S	2 (5)	0 (0)	1 (14)	0 (0)	0 (0)	0 (0)	1 (100)	4 (6)
	R	36 (95)	11 (100)	6 (86)	7 (100)	1 (100)	1 (100)	0 (0)	62 (94)
**CL**	S	1 (3)	0 (0)	0 (0)	0 (0)	0 (0)	0 (0)	0 (0)	1 (2)
	R	37 (97)	11 (100)	7 (100)	7 (100)	1 (100)	1 (100)	1 (100)	65 (98)
**CXM**	S	1 (3)	1 (9)	1 (14)	1 (14)	0 (0)	0 (0)	0 (0)	4 (6)
	R	37 (97)	10 (91)	6 (86)	6 (86)	1 (100)	1 (100)	1 (100)	62 (94)
**CAZ**	S	5 (13)	1 (9)	1 (14)	2 (29)	0 (0)	1 (100)	1 (100)	11 (17)
	R	33 (87)	10 (91)	6 (86)	5 (71)	1 (100)	0 (0)	0 (0)	55 (83)
**FEP**	S	4 (11)	3 (27)	2 (29)	2 (29)	0 (0)	1 (100)	1 (100)	13 (20)
	R	34 (89)	8 (73)	5 (71)	5 (71)	1 (100)	0 (0)	0 (0)	53 (80)
**ETP**	S	1 (3)	1 (9)	1 (14)	0 (0)	0 (0)	0 (0)	1 (100)	4 (6)
	R	37 (97)	10 (91)	6 (86)	7 (100)	1 (100)	1 (100)	0 (0)	62 (94)
**IPM**	S	9 (24)	2 (18)	1 (14)	0 (0)	0 (0)	1 (100)	1 (100)	14 (21)
	R	29 (76)	9 (82)	6 (86)	7 (100)	1 (100)	0 (0)	0 (0)	52 (79)
**MEM**	S	10 (26)	2 (18)	1 (14)	0 (0)	0 (0)	1 (100)	1 (100)	15 (23)
	R	28 (74)	9 (82)	6 (86)	7 (100)	1 (100)	0 (0)	0 (0)	51 (77)
**GM**	S	25 (66)	8 (73)	5 (71)	2 (29)	1 (100)	1 (100)	1 (100)	43 (65)
	R	13 (34)	3 (27)	2 (29)	5 (71)	0 (0)	0 (0)	0 (0)	23 (35)
**SXT**	S	24 (63)	6 (55)	5 (71)	3 (43)	1 (100)	1 (100)	1 (100)	41 (62)
	R	14 (37)	5 (45)	2 (29)	4 (57)	0 (0)	0 (0)	0 (0)	25 (38)
**CIP**	S	6 (16)	2 (18)	4 (57)	0 (0)	1 (100)	1 (100)	1 (100)	15 (23)
	R	32 (84)	9 (82)	3 (43)	7 (100)	0 (0)	0 (0)	0 (0)	51 (77)
**COL**	S	30 (79)	8 (73)	7 (100)	6 (86)	1 (100)	1 (100)	0 (0)	53 (80)
	R	8 (21)	3 (27)	0 (0)	1 (14)	0 (0)	0 (0)	1 (100)	13 (20)

Table S2. Specific primers and conditions used in plasmid replicon typing.

Replicon	Primer name	Primer nucleotide sequence (5' - 3')	Amplicon size (bp)	PCR conditions	Reference
HI1	HI1f	GGAGCGATGGATTACTTCAGTAC	471	Initial denaturation step at 94°C for 5 min; 30 cycles of 94°C for 1 min, annealing temperature: 60°C (52°C for FrepB) for 30 sec; 72°C for 1 min; final extension 72°C for 5 min	[64]
	HI1r	TGCCGTTTCACCTCGTGAGTA			
HI2	HI2f	TTTCTCCTGAGTCACCTGTTAACAC	644		
	HI2r	GGCTCACTACCGTTGTCATCCT			
I1-Iγ	I1f	CGAAAGCCGGACGGCAGAA	139		
	I1r	TCGTCGTTCCGCCAAGTTCGT			
X	Xf	AACCTTAGAGGCTATTTAAGTTGCTGAT	376		
	Xr	TGAGAGTCAATTTTTATCTCATGTTTTAGC			
L/M	L/Mf	GGATGAAAACTATCAGCATCTGAAG	785		
	L/Mr	CTGCAGGGGCGATTCTTTAGG			
N	Nf	GTCTAACGAGCTTACCGAAG	559		
	Nr	GTTTCAACTCTGCCAAGTTC			
FIA	FIAf	CCATGCTGGTTCTAGAGAAGGTG	462		
	FIAr	GTATATCCTTACTGGCTTCCGCAG			
FIB	FIBf	GGAGTTCTGACACACGATTTTCTG	702		
	FIBr	CTCCCGTCGCTTCAGGGCATT			
W	Wf	CCTAAGAACAACAAAGCCCCCG	242		
	Wr	GGTGCGCGGCATAGAACCGT			
Y	Yf	AATTCAAACAACACTGTGCAGCCTG	765		
	Yr	GCGAGAATGGACGATTACAAAACTTT			
P	Pf	CTATGGCCCTGCAAACGCGCCAGAAA	534		
	Pr	TCACGCGCCAGGGCGCAGCC			
FIC	FICf	GTGAACTGGCAGATGAGGAAGG	262		
	FICr	TTCTCCTCGTCGCCAAACTAGAT			
A/C	A/Cf	GAGAACCAAAGACAAAGACCTGGA	465		
	A/Cr	ACGACAAACCTGAATTGCCTCCTT			
T	Tf	TTGGCCTGTTTGTGCCTAAACCAT	750		
	Tr	CGTTGATTACACTTAGCTTTGGAC			
FIIs	FIIsf	CTGTCGTAAGCTGATGGC	270		
	FIIsr	CTCTGCCACAAACTTCAGC			
K	K/Bf	GCGGTCCGGAAAGCCAGAAAAC	160		
	Kr	TCTTTCACGAGCCCGCCAAA			
B/O	K/Bf	GCGGTCCGGAAAGCCAGAAAAC	159		
	B/Or	TCTGCGTTCCGCCAAGTTCGA			
FrepB	FrepBf	TGATCGTTTAAGGAATTTTG	270		
	FrepBr	GAAGATCAGTCACACCATCC			
ColE	oricolEf	GTTCGTGCATACAGTCCA	187	Initial denaturation step at 94°C for 5 min; 30 cycles of 94°C for 1 min, annealing temperature: 57°C for 30 sec; 72°C for 1 min; final extension 72°C for 5 min	[65]
	oricolEr	GGCGAAACCCGACAGGACT			
R	IncRf	TCGCTTCATTCCTGCTTCAGC	251	Initial denaturation step at 94°C for 5 min; 30 cycles of 94°C for 1 min, annealing temperature: 62°C for 30 sec; 72°C for 1 min; final extension 72°C for 5 min	
	IncRr	GTGTGCTGTGGTTATGCCTCA			
U	IncUf	TCACGACACAAGCGCAAGGG	843		
	IncUr	TCATGGTACATCTGGGCGC			
L/M (OXA-48)	RepA-A	GACATTGAGTCAGTAGAAGG	925	Initial denaturation step at 94°C for 2:30 min; 35 cycles of 94°C for 25 sec, annealing temperature: 55°C for 25 sec; 72°C for 1 min; final extension 72°C for 2 min	[66]
	RepA-B	CGTGCAGTTCGTCTTTCGGC			
	TraU-A	ATCTCACGCAATCTTACGTC	577		
	TraU-B	TCGCGTCATGCGTGATCTTC			
	ParA-A	GCAGTCAAAACGTTGATCAG	532		
	ParA-B	GATCGCAATGCGTCTTGGTG			

Table S3. Identification of *Aeromonas* strains by MALDI-TOF MS and/or 16S rRNA gene sequencing. Red letters indicate discrepancies between MALDI-TOF MS and 16S identification. ID represents the isolate designation; TWW and H refer to the source of bacterial isolation, treated municipal wastewater and hospital wastewater, respectively. ND, not determined.

GenBank accession number	ID	MALDI-TOF MS	Score values[1]	Symbol[1]	Consistency[2]	16S identification	Identities
OQ532942	A36 (TWW)	*Aeromonas caviae*	2.212	(++)	B	*Aeromonas caviae*	886/886 (100 %)
OQ532917	A1 (TWW)	*Aeromonas* sp.	1.523	(-)	C	*Aeromonas caviae*	796/799 (99 %)
OQ532944	A4 (TWW)	*Aeromonas caviae*	2.109	(++)	B	*Aeromonas hydrophila*	864/868 (99 %)
OQ532936	A9 (TWW)	*Aeromonas caviae*	2.06	(++)	A	*Aeromonas caviae*	848/858 (99 %)
OQ532945	A11 (TWW)	*Aeromonas caviae*	2.236	(++)	B	*Aeromonas caviae*	863/865 (99 %)
OQ532932	A23 (TWW)	*Aeromonas caviae*	2.172	(++)	A	*Aeromonas hydrophila*	727/727 (100 %)
OQ532946	A3 (TWW)	*Aeromonas caviae*	2.227	(++)	A	*Aeromonas caviae*	865/866 (99 %)
OQ532948	A20 (TWW)	*Aeromonas caviae*	2.117	(++)	A	*Aeromonas caviae*	876/879 (99 %)
OQ532949	A31 (TWW)	*Aeromonas caviae*	2.286	(++)	A	*Aeromonas caviae*	931/940 (99 %)
OQ532955	A6 (TWW)	*Aeromonas caviae*	2.11	(++)	A	*Aeromonas caviae*	869/873 (99 %)
OQ532927	A19 (TWW)	*Aeromonas caviae*	2.213	(++)	B	*Aeromonas caviae*	949/952 (99 %)
OQ532940	A30 (TWW)	*Aeromonas caviae*	2.09	(++)	B	*Aeromonas hydrophila*	883/886 (99 %)
OQ532931	A2 (TWW)	*Aeromonas caviae*	2.18	(++)	B	*Aeromonas caviae*	819/821 (99 %)
OQ532930	A15 (TWW)	*Aeromonas caviae*	2.27	(++)	A	*Aeromonas caviae*	815/819 (99 %)
OQ532935	A32 (TWW)	*Aeromonas caviae*	2.148	(++)	B	*Aeromonas caviae*	874/883 (99%)
OQ532913	A38 (TWW)	*Aeromonas* sp.	1.664	(-)	C	*Aeromonas caviae*	607/607 (100%)
OQ532915	A8 (TWW)	*Aeromonas* sp.	1.672	(-)	C	*Aeromonas caviae*	590/590 (100 %)
OQ532958	A18 (H)	*Aeromonas caviae*	2.156	(++)	A	*Aeromonas caviae*	614/615 (99 %)
OQ532947	A21 (TWW)	*Aeromonas caviae*	2.123	(++)	B	*Aeromonas caviae*	966/969 (99 %)
OQ532938	A12 (TWW)	*Aeromonas caviae*	2.067	(++)	B	*Aeromonas caviae*	873/876 (99 %)
OQ532933	A35 (TWW)	*Aeromonas caviae*	2.039	(++)	A	*Aeromonas caviae*	865/865 (100 %)
OQ532934	A37 (TWW)	*Aeromonas caviae*	2.087	(++)	B	*Aeromonas caviae*	856/857 (99 %)
OQ532941	A29 (TWW)	*Aeromonas caviae*	2.019	(++)	A	*Aeromonas caviae*	935/940 (99 %)
OQ532952	A28 (TWW)	*Aeromonas caviae*	2.264	(++)	B	*Aeromonas caviae*	864/865 (100 %)
OQ532953	A10 (TWW)	*Aeromonas caviae*	2.24	(++)	B	*Aeromonas caviae*	882/884 (99 %)
OQ532961	A24(TWW)	*Aeromonas caviae*	2.028	(++)	B	*Aeromonas caviae*	864/869 (99 %)
OQ532920	A14 (TWW)	*Aeromonas* sp.	1.465	(-)	C	*Aeromonas caviae*	755/756 (99 %)
	A22 (TWW)	*Aeromonas caviae*	2.122	(++)	B	ND	
	A26 (TWW)	*Aeromonas caviae*	2.031	(++)	B	ND	
OQ532960	A34 (TWW)	*Aeromonas caviae*	2.366	(++)	B	*Aeromonas caviae*	870/878 (99 %)
	A5 (TWW)	*Aeromonas caviae*	2.096	(++)	B	ND	
OQ532925	A13 (TWW)	*Aeromonas* sp.	1.61	(-)	C	*Aeromonas caviae*	909/915 (99 %)
	A17 (TWW)	*Aeromonas caviae*	2.151	(++)	B	ND	
	A33 (TWW)	*Aeromonas caviae*	2.067	(++)	A	ND	
OQ532924	A27 (TWW)	*Aeromonas* sp.	1.669	(-)	C	*Aeromonas caviae*	910/914 (99%)
	A7 (TWW)	*Aeromonas caviae*	2.166	(++)	B	ND	
OQ532911	A16 (TWW)	*Aeromonas* sp.	1.68	(-)	C	*Aeromonas caviae*	533/533 (100 %)
	A25 (TWW)	*Aeromonas caviae*	2.259	(++)	A	ND	
OQ532956	A39 (TWW)	*Aeromonas hydrophila*	2.058	(++)	A	*Aeromonas hydrophila*	870/876 (99 %)
OQ532957	A40 (TWW)	*Aeromonas hydrophila*	2.014	(++)	B	*Aeromonas hydrophila*	882/88 8 (99 %)
OQ532943	A42 (TWW)	*Aeromonas hydrophila*	2.03	(++)	B	*Aeromonas hydrophila*	869/871 (99 %)
OQ532950	A45 (TWW)	*Aeromonas hydrophila*	2.238	(++)	B	*Aeromonas hydrophila*	949/955 (99 %)
OQ532928	A48 (TWW)	*Aeromonas hydrophila*	2.169	(++)	B	*Aeromonas hydrophila*	948/953 (99 %)
OQ532939	A49 (TWW)	*Aeromonas hydrophila*	2.062	(++)	A	*Aeromonas hydrophila*	935/940 (99 %)
OQ532954	A41 (TWW)	*Aeromonas hydrophila*	2.199	(++)	A	*Aeromonas hydrophila*	884/889 (99 %)
OQ532923	A46 (TWW)	*Aeromonas* sp.	1.676	(-)	C	*Aeromonas hydrophila*	916/916 (100 %)
	A47 (TWW)	*Aeromonas hydrophila*	2.15	(++)	B	ND	
	A44 (TWW)	*Aeromonas hydrophila*	2.281	(++)	A	ND	
	A43 (H)	*Aeromonas hydrophila*	2.143	(++)	B	ND	
OQ532916	A51 (TWW)	*Aeromonas sp.*	1.617	(-)	C	*Aeromonas media*	800/800 (100 %)
OQ532918	A50 (TWW)	*Aeromonas sp.*	1.652	(-)	C	*Aeromonas media*	873/875 (99 %)
OQ532937	A56 (TWW)	*Aeromonas media*	2.021	(++)	A	*Aeromonas media*	950/950 (100 %)
OQ532951	A55 (TWW)	*Aeromonas media*	2.035	(++)	A	*Aeromonas media*	884/886 (99 %)
OQ532959	A54 (TWW)	*Aeromonas media*	2.018	(++)	A	*Aeromonas media*	785/787 (99 %)
OQ532922	A52 (TWW)	*Aeromonas sp.*	1.512	(-)	C	*Aeromonas media*	755/755 (100 %)
OQ532921	A53 (TWW)	*Aeromonas sp.*	1.51	(-)	C	*Aeromonas media*	605/605 (100 %)

Table S3. Continued

GenBank accession number	ID	MALDI-TOF MS	Score values	Symbol	Consistency	16S identification	Identities
OQ532912	A63 (TWW)	*Aeromonas* sp.	1.401	(-)	C	*Aeromonas veronii*	616/616 (100 %)
OQ532919	A60 (H)	*Aeromonas* sp.	1.623	(-)	C	*Aeromonas veronii*	806/806 (100 %)
	A62 (H)	*Aeromonas veronii*	2.191	(++)	B	ND	
	A57 (H)	*Aeromonas veronii*	2.31	(++)	A	ND	
	A58 (H)	*Aeromonas veronii*	2.181	(++)	B	ND	
	A59 (H)	*Aeromonas veronii*	2.091	(++)	B	ND	
OQ532914	A61 (TWW)	*Aeromonas* sp.	1.618	(-)	C	*Aeromonas veronii*	519/519 (100 %)
OQ532929	A64 (TWW)	*Aeromonas salmonicida*	2.188	(++)	B	*Aeromonas media*	821/824 (99 %)
OQ532926	A65 (TWW)	*Aeromonas* sp.	1.632	(-)	C	*Aeromonas rivipollensis*	668/671 (99 %)
	A66 (TWW)	*Aeromonas eucrenophila*	2.072	(++)	B	ND	

[1]Meaning of score values

Range	Interpretation	Symbols
2.300-3.000	High probable species identification	(+++)
2.000-2.299	Secure genus identification, probable species identification	(++)
1.700-1.999	Probable genus identification	(+)
0.000-1.699	Not reliable identification	(-)

[2]Meaning of consistency categories

Category	Description
A	Species consistency
B	Genus consisteny
C	No consistency
